# Nanotherapy for bone repair: milk-derived small extracellular vesicles delivery of icariin

**DOI:** 10.1080/10717544.2023.2169414

**Published:** 2023-01-30

**Authors:** Xinxin Yu, Ming Dong, Lina Wang, Qian Yang, Long Wang, Wenqing Han, Juhong Dong, Tingjiao Liu, Ying Kong, Weidong Niu

**Affiliations:** a School of Stomatology, Dalian Medical University, Dalian, Liaoning, China; b Department of Basic Science of Stomatology, Shanghai Stomatological Hospital, Fudan University, Shanghai, China; c Shanghai Key Laboratory of Craniomaxillofacial Development and Diseases, Fudan University, Shanghai, China; d Department Biochemistry and Molecular Biology, College of Basic Medical Sciences, Dalian Medical University, Dalian, Liaoning, China

**Keywords:** Bone remodeling, icariin, milk, small extracellular vesicles, nanotherapy

## Abstract

Icariin (ICA) played an important role in the treatment of inflammatory bone defects. However, pharmacokinetic studies have shown that its poor bioavailability limited its application. In this context, we isolated bovine milk-derived sEV and prepared sEV-ICA to improve the osteogenic effect of ICA. In this study, we successfully constructed sEV-ICA. sEV-ICA was found to have significantly higher osteogenic efficiency than ICA in cell culture and cranial bone defect models. Mechanistically, bioinformatics analysis predicted that signal transducers and activators of transcription 5 (STAT5a) may bind to the GJA1 promoter, while luciferase activity assays and chromatin immunoprecipitation (ChIP) experiments confirmed that STAT5a directly binded to the GJA1 promoter to promote osteogenesis. We proved that compared with ICA, sEV-ICA showed a better effect of promoting bone repair in vivo and in vitro. In addition, sEV-ICA could promote osteogenesis by promoting the combination of STAT5a and GJA1 promoter. In summary, as a complex drug delivery system, sEV-ICA constituted a rapid and effective method for the treatment of bone defects and could improve the osteogenic activity of ICA.

## 1. Introduction

Bone defects can be formed for many reasons, such as trauma, tumor, inflammation, etc., while advanced resorption, damage and tooth loss in the alveolar bone due to periapical infection can lead to severe bone defects in the craniomaxillofacial region, resulting in deformity and dysfunction (Kinoshita & Maeda, 2013). With growing research on traditional Chinese medicine, studies have shown that icariin (ICA) might play an important role in bone remodeling by promoting bone formation and inhibiting inflammation (Wang et al., 2018). The pharmacological database and analysis platform of the traditional Chinese medicine system (TCMSP) also shows that the bioavailability of ICA is low at approximately 41.58% (Xu et al., 2017). Drug delivery has been a hot topic of research for many years, and researchers are envisaging fine designs to provide drug delivery vehicles (Li et al., 2021; Tian et al., 2022). Therefore, to change the hydrophobicity and utility of ICA, researchers have investigated nanomaterials to load ICA, which may lead to toxicity and biocompatibility problems. Therefore, a more advantageous and cost-effective natural drug delivery system is required to load ICA.

Extracellular vesicles (EVs), which include exosomes (40–150 nm), microvesicles (100–1,000 nm), and apoptotic bodies (1–5 μm), are derived from most types of nanoscale membrane vesicles (Abels & Breakefield, 2016; Shao et al., 2018). EVs are important for intercellular communication and play a vital role in physiological and pathological processes (Grange et al., 2019). As it is difficult to classify EVs according to their biological mechanisms, a nomenclature based on size has been adopted which defines exosomes as small EVs (sEV), and microbubbles as large EVs (Lim et al., 2021). Because of their unique source, structure, and physiological function, EVs are often used as ideal natural endogenous nano-delivery systems for the delivery of endogenous or exogenous drugs (Herrmann et al., 2021). EVs have been tested for the delivery of various therapeutics, including small-molecule drugs, miRNAs, and siRNAs (Faruqu et al., 2018; Busatto et al., 2019; Groot & Lee, 2020). Milk is not only considered an important source of nutrition for infants, but also contains many sEV (Tome-Carneiro et al., 2018). Milk-derived sEV have great potential as carriers for hydrophilic and lipophilic drugs. For example, compared with free drugs, the efficacy of milk-derived paclitaxel (sEV-PAC) against lung tumor xenografts in vivo is significantly higher (Agrawal et al., 2017). Milk-derived EVs are an attractive prospect owing to their high cost-effectiveness, good biocompatibility, high stability, strong targeting ability of ligands, and the ability to carry various forms of therapeutic drugs (Vashisht et al., 2017; Zempleni et al., 2017; Sanwlani et al., 2020). Thus, in this study, we extracted sEV from milk, used milk-derived sEV to carry ICA, and observed whether they can improve the osteogenic effect of ICA and provide a new research direction for the clinical treatment of bone defects.

## 2. Material and methods

### 2.1. Isolation and characterization of milk-derived sEV

Cow milk was collected from healthy, early lactating cows of the Sanhuan ranch (Dalian, CHN). The animals were fed with similar diet and maintained in the same herd. The cows used in this study had moderate activity levels and were free of any diseases. After defatting, the milk was centrifuged at 13,000 × g for 0.5 hours, and then at 100,000 × g for 2 h. Fragments, milk fat globules, casein, and the supernatant were centrifuged at 135,000 × g for 1.5 h to granulate sEV, which were further purified and collected by centrifugation at 100,000 × g for 2 h and 1 h. The protein content of concentrated sEV was measured using a BCA protein assay kit (Beyotime Institute of Biotechnology, Jiangsu, CHN). TEM (GEM-2000EX) was used to directly observe the size and morphology of sEV. NTA was performed to analyze the absolute size distribution of sEV using ZetaView® BASIC NTA (Particle Metrix GmbH, Munich, GER). In addition, sEV were identified by western-blot with anti-CD81 (1:1,000, Abcam, Cambridge, MA, USA), anti-CD63 (1:1,000, Abcam), anti-CD40 (1:1,000, Bioss Antibodies, Woburn, MA, USA), and anti-ALIX (1:1,000, Abbexa Ltd., Cambridge, UK) antibodies. Characterization of sEV was performed following the Minimal Information for Studies of Extracellular Vesicles 2018 (MISEV2018) recommendations by the International Society for Extracellular Vesicles (ISEV).

### 2.2. Drug encapsulation

Drug loading of ICA (Chengdu Must Bio-technology Co. Ltd, Chengdu, CHN) was achieved by mixing the test agent with a suspension of sEV at a ratio of 1: 9 at 22 °C for 24 h. Unbound drug was removed by low-speed centrifugation (10,000 × g) for 10 min, and the drug-loaded sEV were collected by centrifugation at 135,000 × g for 2 h. The pellet was suspended in PBS and stored at −80 °C. Drug loading was determined by spectrophotometric analysis of the drug and HPLC; the protein in the sample and percentage drug load were calculated.

### 2.3. HPLC analysis

The percentage of drug load was determined using HPLC (Shimadzu Corp, Kyoto, JPN). Briefly, acetonitrile (250 μL) was added to drug-loaded sEV (50 μL) to extract the drug and precipitate sEV proteins. The control solution was spiked with a known concentration of ICA and treated with acetonitrile. An Agilent 1,200 liquid chromatography system (Agilent Technologies, Santa Clara, USA) and an API 3,200 liquid chromatography triple quadrupole mass spectrometer were used for analysis.

### 2.4. Cell culture

MC3T3-E1 cells (American Type Culture Collection, Manassas, VA, USA) were grown in α-MEM (Gibco; Thermo Fisher Scientific, Waltham, MA, USA) supplemented with 10% fetal bovine serum (FBS) (AusGeneX, Brisbane, AUS), 100 U/mL penicillin, and 100 mg/mL streptomycin. The cells were maintained at 37 °C and 5% CO_2_ in humidified air. An in vitro model of osteoblasts was established by using MC3T3-E1 cells. MC3T3-E1 cells in the logarithmic growth phase were cultured in α-MEM containing osteogenic induction solution (dexamethasone, 10 nmol/L; β-sodium glycerophosphate, 10 mmol/L; and vitamin C, 50 μg/mL).

### 2.5. Immunofluorescence (IF)

After seeding into 24-well plates, the MC3T3-E1 cells were treated with culture medium containing sEV and sEV-ICA with the PKH67 marker (Sigma-Aldrich, Merck KGaA, Darmstadt, GER). Cells were fixed in 4% paraformaldehyde for 10 min, permeabilized with 0.25% Triton X-100 for 10 min, and blocked with 1% FBS albumin for 30 min. The cells were incubated with an anti-phalloidin antibody (1:200; Yeasen Biotechnology, Shanghai, CHN) overnight at 4 °C, the slides were counterstained with phenylindole (1:3,000, Yeasen Biotechnology) to stain the cell nuclei. Stained cells were observed under a fluorescence microscope (Olympus, Tokyo, JPN).

### 2.6. CCK-8 assay

Cells were seeded in 96-well plates at 2 × 10^3^ cells/well and treated with culture medium containing ICA. Proliferation was measured using Cell Counting Kit-8 (CCK-8; APExBIO, Houston, TX, USA). For the quantitative analysis of cell proliferation, 10 μL of CCK-8 solution was added to each well. Two hours later, absorbance was measured at 450 nm using a spectrophotometer.

### 2.7. ALP activity

MC3T3-E1 cells in the logarithmic growth phase, osteogenic medium were added, supplemented with ICA, sEV, or sEV-ICA at the optimal concentration, as determined above. The cellular ALP activity of the cultures was measured on day five. Protein concentrations were determined using a bicinchoninic acid protein assay kit. ALP activity was measured using an ALP assay kit (Nanjing Jiancheng Bioengineering Institute, Nanjing, CHN). Enzyme activity was normalized against protein concentration and expressed as U/g protein.

### 2.8. Real-time qPCR assays

Samples were added to TRIzol Reagent (Sangon Biotech, Shanghai, CHN) to extract the total RNA. The reverse transcription reaction was performed in a volume of 20 μL with an Evo M-MLV RT Kit with gDNA Clean for qPCR (Accurate Biotechnology Ltd., Shenzhen, CHN). which was prepared according to the SYBR Green Premix Pro Taq HS qPCR Kit (Accurate Biotechnology). The data were analyzed using the 2^−ΔΔCt^ method. Real-time qPCR reaction contained SYBR® Premix Ex Taq™ II (TaKaRa, Tokyo, JPN) (12.5 μL), primer mix (2 μL), cDNA (1 μL), and deionized water (9.5 μL) in a final total volume of 20 μL. All PCRs were performed for 40 cycles and run-in triplicate.

### 2.9. Western-blot

Total protein was extracted using RIPA buffer. Protein concentration was measured using a BCA protein assay kit (Beyotime Biotechnology). The membranes were incubated overnight at 4 °C with specific anti-RUNX2 (1:500, Signalway Antibody, State of California, USA), anti-ALP (1:1,000, Abcam), anti-STAT5a (1:1,000, Cell Signaling Technology, Massachusetts, USA), anti-GJA1 (1:1,000, Bioworld, Visalia, USA), and anti-GAPDH (diluted 1:5,000, Bioworld) antibodies. Incubation with secondary antibody (diluted 1:5,000, ABclonal, Wuhan, CHA) for 1 h. The ECL luminescent solution was configured to collect the blotting results with a Bio-Rad gel imaging system, and the results were analyzed with Image Lab software.

### 2.10. The analysis of sEV and sEV-ICA release

sEV (300 μg) and sEV-ICA (300 μg) were resuspended in filtered 5% GelMA (80 μL) (Engineering for Life, Suzhou, CHN), and photo-crosslinked via exposure to 9.16-W/cm^2^ UV light for 20 s using an Omnicure S2000 (Lumen Dynamics Group, Waltham, MA, USA). The hydrogels were immersed in PBS in a 24-well plate. At specific time points, the surface supernatant was collected and fresh PBS was added. The released sEV and sEV-ICA were quantified and expressed as release percentages.

### 2.11. Animal experiments

All animals were provided by Dalian Medical University. The experimental protocol was approved by the Institutional Review Board of the School of Stomatology, Dalian Medical University (2021006) and kept in an animal control facility with a light/dark cycle of 12 h and a temperature of 22 ± 2 °C.

Construction of a skull defect model: Twenty male C57BL/6 mice (aged 8 weeks) were divided into five groups (*n* = 4). The mice were anesthetized with pentobarbital sodium (30 mg/kg), an arcuated incision was made on the scalp, and the skull was exposed. A 1.5 × 1.5 mm defect was created using a high-speed drill with a dental slow grinder (Saeshin, Daegu, Kr). Animals in groups 1–4 were implanted with composites composed of GelMA treated with PBS, ICA (400 μg), sEV (3,200 μg), or sEV-ICA (3,200 μg). Group 5 received no implants and served as the negative control group. The cranial bones were harvested two weeks after implantation.

### 2.12. Histological evaluation of regenerated bone tissue

Experimental samples were decalcified at 22 °C for 28 days with 10% EDTA and washed three times with PBS. Serial sections (8 μm) were prepared from the tissues. The experimental tissues were subsequently embedded in paraffin and stained with hematoxylin and eosin (HE) trichrome. A working solution of normal goat serum was added and the sections were incubated for 60 min at 22 °C. The samples were probed overnight at 4 °C with anti-BMP2 (1:200; Abcam). Positive cells were counted under an Olympus BX43 light microscope. The mean number of positive cells in five consecutive sections was recorded for each specimen.

### 2.13. Short interfering RNA (siRNA) knockdown experiments

MC3T3-E1 cells were seeded in 35 mm plates (1 × 10^6^ cells). In gene knockout experiments, siRNA (50 nmol/well) transfected with GJA1, STAT5a, Nrf2 and PAX6 genes and negative control siRNA (General boil, Anhui, China) were used. To perform overexpression experiments, cells were transfected with a STAT5a overexpression vector (over-STAT5a plasmid; 200 nmol/well). MC3T3-E1 cells were transfected using Lip3000 (Thermo Fisher Scientific). Transfection efficiency was evaluated using fluorescence quantitative PCR.

### 2.14. Transcriptome sequencing and analysis

RNA sequencing was performed by Novogene Technology Co., Ltd (Shanghai, CHN). Illumina sequencing platform was used to read high quality sequencing data quickly and efficiently. The number of reads covered from initiation to termination of each gene was counted using the Subread software. HISAT2 software was used to accurately compare clean reads with GRCm39 mice genome to obtain the location information of the reads.

### 2.15. Luciferase reporter assay

The luciferase reporter assay was performed in 293 T cells. Cells were seeded in 35 mm plates (1 × 10^6^ cells) and allowed to grow for 24 h. DNA fragments encoding mice GJA1 promoters were ligated into pEZX-PG04.1 (GeneCopoeia Inc., Rockville, MD, USA) truct GJA1 promoter-luciferase reporter systems, pEZX-PG04.1 Vector was used as control group (Con-GAJ1). The STAT5a fragment was constructed on the pReceiver-M02 vector (Over-STAT5a) (GeneCopoeia Inc., Rockville), which served as the control group (Con-STAT5a). Cells were then transfected in triplicate with one of the four vectors (GJA1-pomoter, Con-GJA1, Over-STAT5a, and Con-STAT5a) using Lipopectamine-3000 (Thermo Fisher Scientific). Gaussia luciferase (GLuc) activity and SELP activity were assayed 48 h after transfection using the Secrete-Pair Dual Luminescence Assay Kit (GeneCopoeia) according to the manufacturer’s instructions.

### 2.16. Chip assay

MC3T3-E1 cells were plated into 15 cm dishes (1.5 × 10^7^ cells) at the densities described above in the Cell Culture section. ChIP was performed using antibodies against STAT5a (Cell Signaling Technology) (156 μg/mL) or rabbit IgG (Cell Signaling Technology) (2.5 mg/mL) using the ChIP-IT Express Enzymatic (Active Motif, Carlsbad, CA) according to the manufacturer’s instructions. DNA was amplified using primers flanking the putative STAT5a binding site (PROMO) within the GJA1 promoter.

### 2.17. Statistical analysis

All data were subjected to statistical analysis without any transformation or normalization and are expressed as the mean standard deviation (SD). The sample size (n) is described in the Experimental section and in the figure captions. Statistical tests and data illustrations were performed using GraphPad Prism version 8.0 (GraphPad Software, San Diego, CA, USA). Significance was analyzed by Mann–Whitney test. Statistical significance was set at *P* < .05.

## 3. Results

### 3.1. Preparation and identification of milk sEV and sEV-ICA

**Figure 1.**
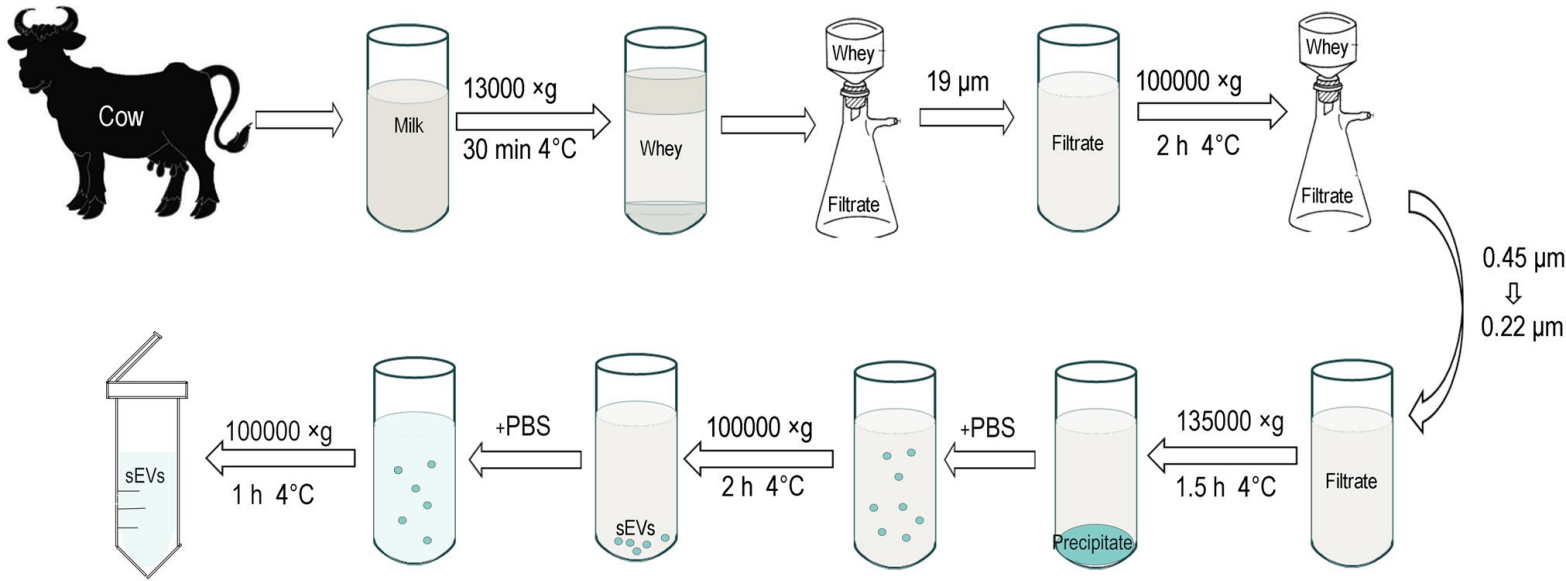
The separation process of milk sEV.

**Figure 2.**
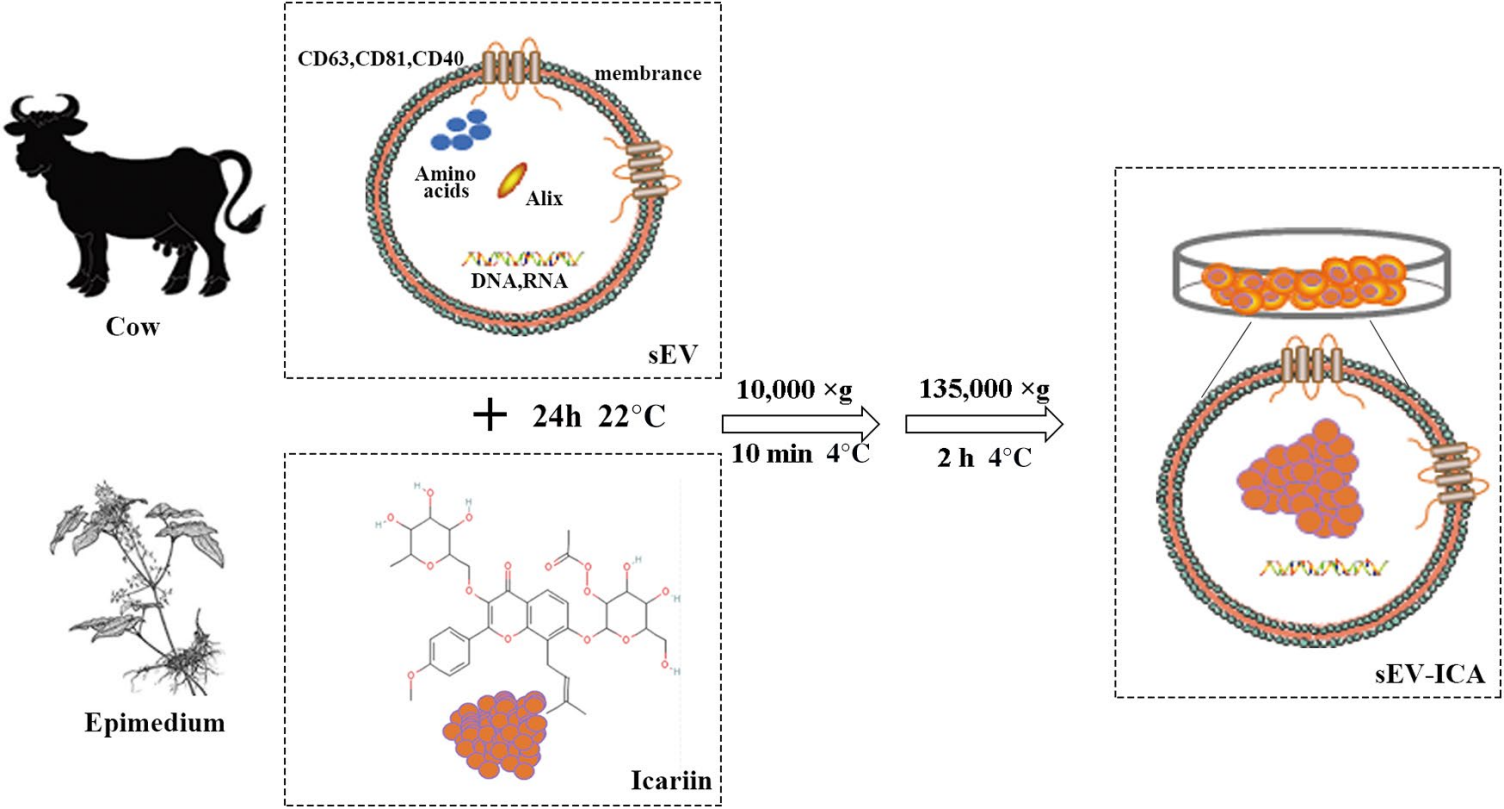
Preparation of sEV-ICA by co-incubation.

**Figure 3.**
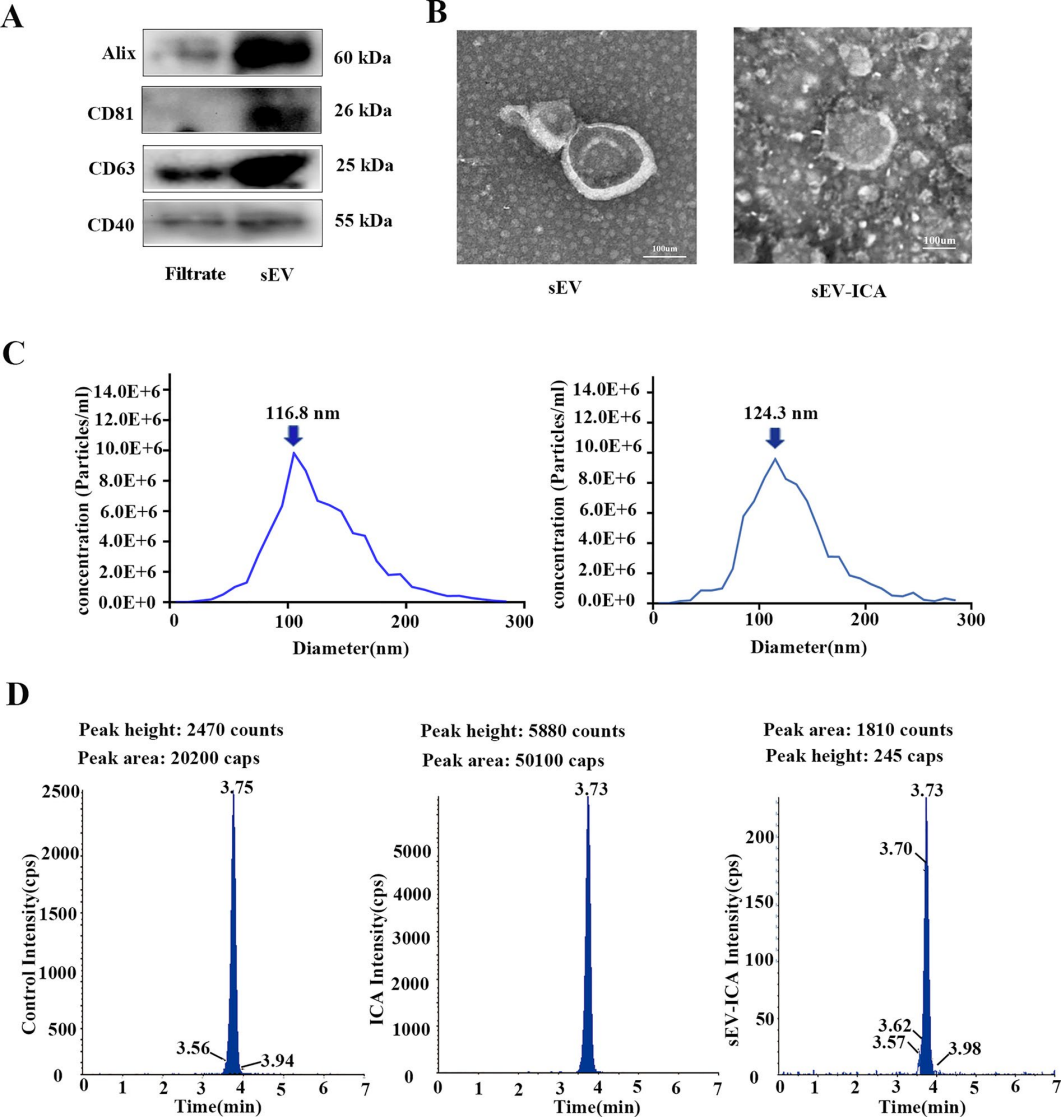
Preparation and identification of sEV and sEV-ICA. (A) Western-blot was conducted to analyze protein expression of the exosome markers CD63, CD81, and ALIX and the microcapsule surface marker CD40 in different batches of milk-derived sEV. (B) Observation of the structure of milk-derived sEV and sEV-ICA using transmission electron microscopy (TEM), and the peak analysis of sEV and sEV-ICA (concentration) was shown in the table. (C) Nanoparticle tracking analysis detection of the median particle size of milk-derived sEV and sEV-ICA. (D) High performance liquid chromatography indicating the extent of ICA incorporation into milk-derived sEV-ICA.

Extraction of milk-derived sEV by ultracentrifugation (Figure 1). Drug loading of ICA was achieved by mixing the test agent with a suspension of sEV at a ratio of 1:9 at 22 °C for 24 h (Figure 2). Western-blot results showed that milk-derived sEV were positive for the expression of the membrane markers tetraspanin CD81, CD63, and Alix, whereas the microbubble surface marker CD40 was not expressed (Figure 3A). Transmission electron microscopy showed that milk-derived sEV had a typical lipid bilayer structure with a size between 30 and 150 nm, the co-incubation drug-loading method did not change the lipid bilayer structure or the size of sEV (Figure 3B). The results of nano-particle tracking analysis showed that the average particle size of milk-derived sEV was 116.8 nm, and the average particle size of sEV-ICA was 124.3 nm. After 25,000 times dilution, the particle concentration of sEV was 6.9 × 10^6^ particles/ml, while after 15,000 times dilution, the particle concentration of sEV-ICA was 7.9 × 10^6^ particles/mL (Figure 3C). Analysis of the drug loading level of ICA in the sEV-ICA preparation by high-performance liquid chromatography (HPLC) revealed that the level was between 5% and 8% (Figure 3D). These results show that we can successfully isolate sEV and successfully prepare sEV-ICA by co-incubation.

### 3.2. Milk sEV-ICA enhanced the effect of ICA on proliferation and differentiation of osteoblasts

**Figure 4.**
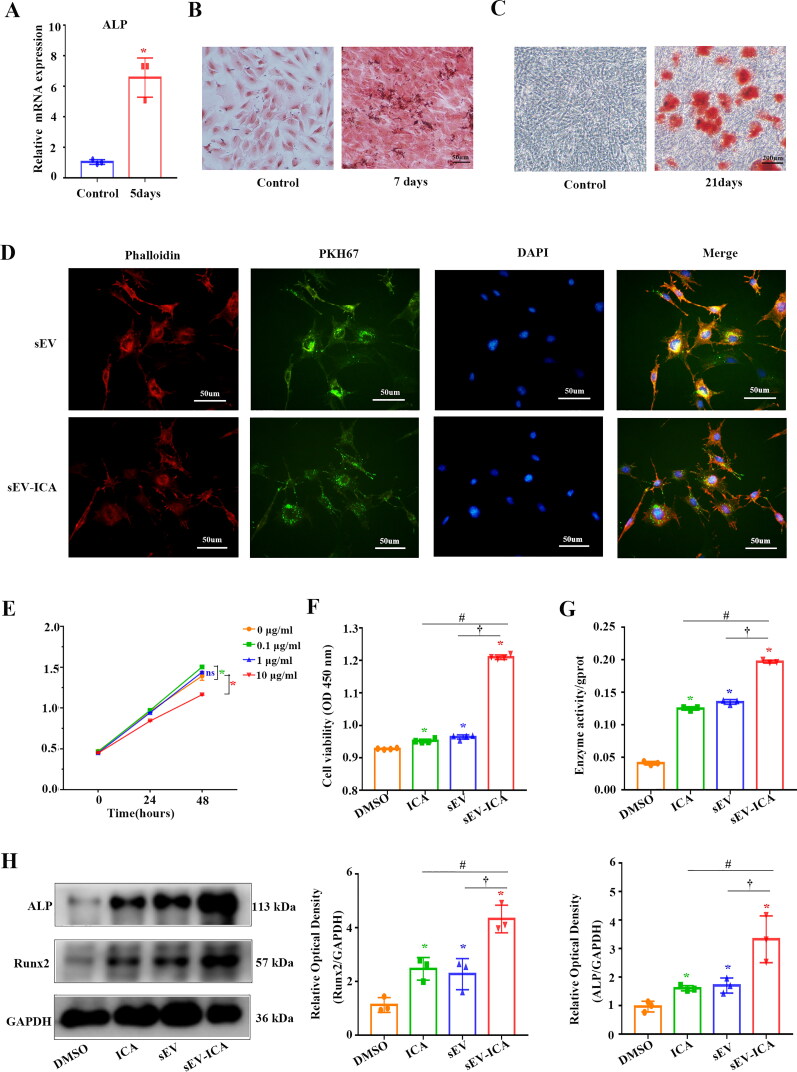
sEV-ICA promote osteoblast proliferation and differentiation. (A) The expression level of ALP was analyzed by real-time qPCR after 5 days of induction (n = 3). Statistical significance assessed by Mann-Whitney test, *P-value < .05. (B) After 7 days of induction, MC3T3-E1 cells were stained with alkaline phosphatase. (C) After 21 days of induction, MC3T3-E1 cells were stained with Alizarin red. (D) Fluorescence staining showing that phalloidin stained the cytoskeleton red, PKH67 stained the exosome membranes green, and DAPI stained the cell nuclei blue (scale bar = 50 μm). (E) Proliferative activity was determined after 0, 24 and 48 h by CCK8 assays (n = 3). Statistical significance assessed by Mann-Whitney test, ns: no significant difference, *P-value < .05. (F) CCK8 detection of proliferation ability (n = 3). Statistical significance assessed by Mann-Whitney test, ^*,#,†^*P*-value < .05. (G) Alkaline phosphatase detection of differentiation ability (n = 3). Statistical significance assessed by Mann-Whitney test, ^*,#,†^*P*-value < .05. (H) The expression of osteogenic marker proteins was detected by western-blot (n = 3). Statistical significance assessed by Mann-Whitney test, ^*,#,†^*P*-value < .05.

Alkaline phosphatase (ALP) expression in the mice preosteoblastic cell line (MC3T3-E1 cells) induced for 5 days with sEV-ICA was significantly higher than that in uninduced cells (Figure 4A). After 7 days of induction, ALP staining showed that ALP activity increased significantly (Figure 4B). After 21 days of induction, many dark-red calcified nodules were observed on the cell surface (Figure 4C), indicating that the MC3T3-E1 cells were successfully induced. To determine the uptake of sEV in vitro, the membrane dye PKH67 is commonly used to label sEV, while phalloidin is used to label the cytoskeleton^17,18^. Mice MC3T3-E1 cells were treated with PKH67-labeled sEV and sEV-ICA for 12 h. We observed that PHK67-labeled sEV and sEV-ICA coexisted uniformly in cells (Figure 4D). First, we studied the effects of different concentrations of ICA on the proliferation of MC3T3-E1 cells. Cell Counting Kit-8 (CCK8) results showed that 0.1 μg/mL ICA treatment significantly promoted the proliferation of MC3T3-E1 (Figure 4E). This study first examined the individual effects of DMSO, ICA (0.1 μg/mL), sEV (with equal amounts of sEV in the sEV-ICA group) and sEV-ICA (containing 0.1 μg/mL ICA based on 5% loading rate) on the proliferative activity of MC3T3-E1 cells by CCK-8 assay. Compared with other groups, sEV-ICA group significantly promoted cell proliferation. ALP activity was used as an osteoblast differentiation marker in MC3T3-E1 cells (Figure 4F). After MC3T3-E1 cells were treated with DMSO, ICA, sEV and sEV-ICA for 48 hours, the ALP activity of sEV-ICA group was significantly higher than that of other groups (Figure 4G). Western-blot results showed that the expressions of osteogenic markers ALP and Runx2 were significantly increased in sEV-ICA group (Figure 4H). These results suggested that sEV-ICA may promote the early stage of osteoblast differentiation in MC3T3-E1 cells.

### 3.3. sEV-ICA enhanced the bone regeneration ability of ICA in mice skull defect model

**Figure 5.**
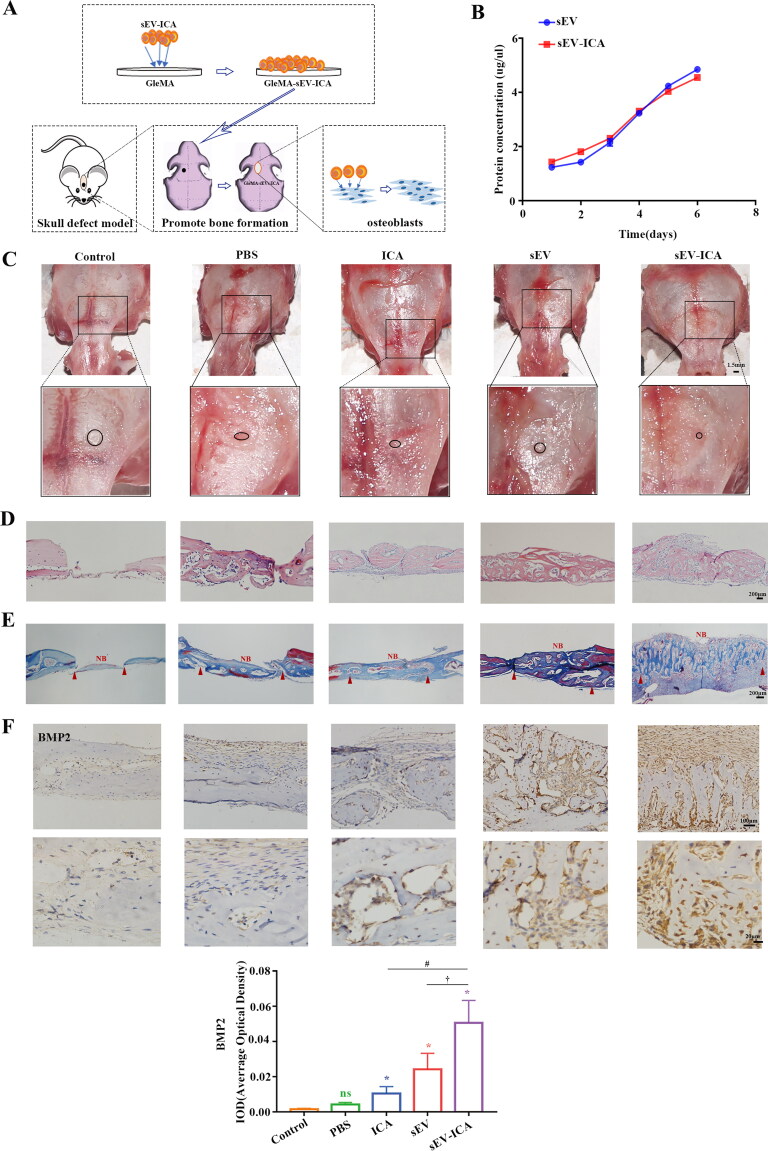
sEV-ICA promote bone repair of skull defects in mice. (A) Schematic diagram of the mouse skull defect model. (B) Profile of sEV released from the hydrogels (n = 3). (C) Skull condition two weeks after surgery. (D) HE trichrome staining of the mice defect implanted with the drug (scale bar = 200 μm). (E) Immunohistochemical staining of BMP2 in calvarial defect tissue of mice (n = 4/group) (scale bar = 100 μm, 20 μm). Statistical significance assessed by Mann-Whitney test, ns: no significant difference, ^*,#,†^*P*-value < .05.

To explore the reparative effect of sEV-ICA in mice skull defect, sEV-ICA was loaded into a gelatin methacryloyl (GelMA) hydrogel, implanted into mice skull defect, and histological analysis was performed two weeks after the operation (Figure 5A). As shown by the sEV and sEV-ICA release curve, sEV and sEV-ICA were effectively encapsulated in the GelMA hydrogel and displayed representative long-term sustained-release behavior (Figure 5B). A bone defect of 1.5 × 1.5 mm was formed in the mice skulls^19^. Two weeks after the operation, the defect was completely closed in the sEV-ICA group, whereas in the other groups, it was not (Figure 5C). Hematoxylin and eosin (H&E) staining (Figure 5D) revealed much new bone formation. Immunohistochemical staining and statistical analysis of bone morphogenetic protein 2 (BMP2) expression (Figure 5E) revealed that BMP2 expression was enhanced in the sEV-ICA group compared to the other groups. The results suggest that sEV-ICA enhances the ability of ICA to promote bone repair of skull defects in mice.

### 3.4. Transcriptome analysis of osteoblasts treated with mlik sEV-ICA

**Figure 6.**
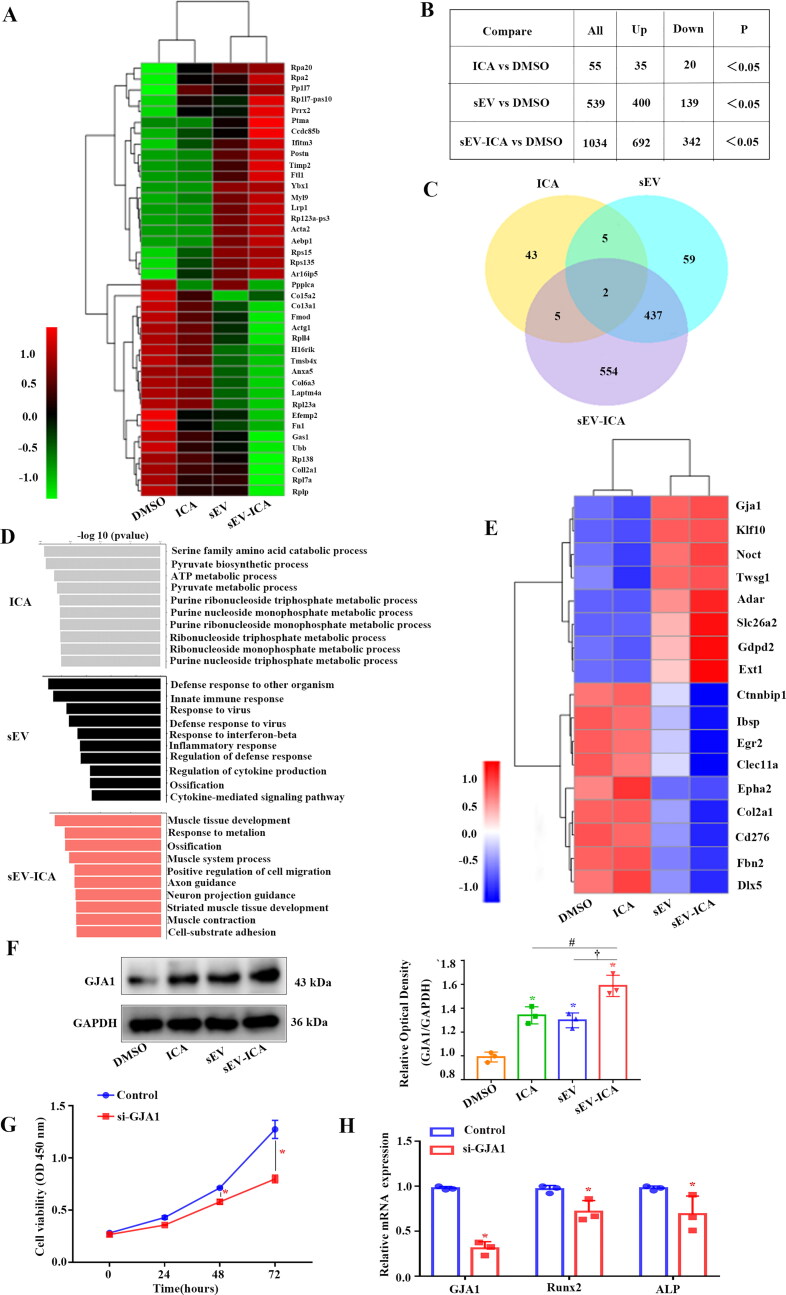
Transcriptome analysis of sEV-ICA on mC3T3-E1 cells. (A) Heatmap of RNA sequencing data. (B) Up-regulated and down-regulated genes in the three comparison groups (ICA vs. DMSO, sEV vs. DMSO, sEV-ICA vs. DMSO). (C) Venn diagram. (D) Enriched Biological Processes with GO term using differentially expressed genes are listed. (E) RNA Sequencing Heat Map Analysis of ossification Related Factors. (F) Expression of GJA1 in MC3T3-EI treated with DMSO, ICA, sEV, and sEV-ICA, as assessed by western-blot (n = 3). Statistical significance assessed by Mann-Whitney test, ^*,#,†^*P*-value < .05. (G) CCK-8 assay showed the proliferation of MC3T3-E1 cells after down-regulation of GJA1 (n = 3). Statistical significance assessed by Mann-Whitney test, *P-value < .05. (H) Real-time qPCR analysis showed the expression of the osteogenesis-related factors ALP and Runx2 after down-regulation of GJA1(n = 3). Statistical significance assessed by Mann-Whitney test, *P-value < .05.

To gain insight into the mechanism by which sEV-ICA promoted bone formation, we first examined the changes that occur at the transcriptome level in dimethyl sulfoxide (DMSO)-, ICA-, sEV -, and sEV-ICA-treated MC3T3-E1 cells via RNA sequencing, producing a heat map of relevant differentially expressed genes (DEGs) (Figure 6A). According to the criteria for screening DEGs, the identified DEGs compared to the control group are shown in Figure 6(B). A Venn diagram showed that compared with the control group, there were 43 DEGs in the ICA group, 59 DEGs in the sEV group, and 554 DEGs in the sEV-ICA group (Figure 6C). Gene Ontology (GO) analysis of the DEGs showed that compared with other groups, the DEGs identified in the sEV-ICA group were mainly involved in muscle tissue development and ossification (Figure 6D). A heatmap of ossification-related DEGs (Figure 6E) showed changes in GJA1 expression, and the reliability of gene sequencing was verified by western-blot (Figure 6F). Downregulation of GJA1 expression inhibited the proliferation of MC3T3-E1 cells (Figure 6G), whereas the expression levels of the osteogenesis-related factors ALP and Runx2 also decreased (Figure 6H). These results showed that GJA1 plays an important role in the proliferation and differentiation of osteoblasts.

### 3.5. Milk sEV-ICA activated GJA1 transcription by upregulating STAT5a

**Figure 7.**
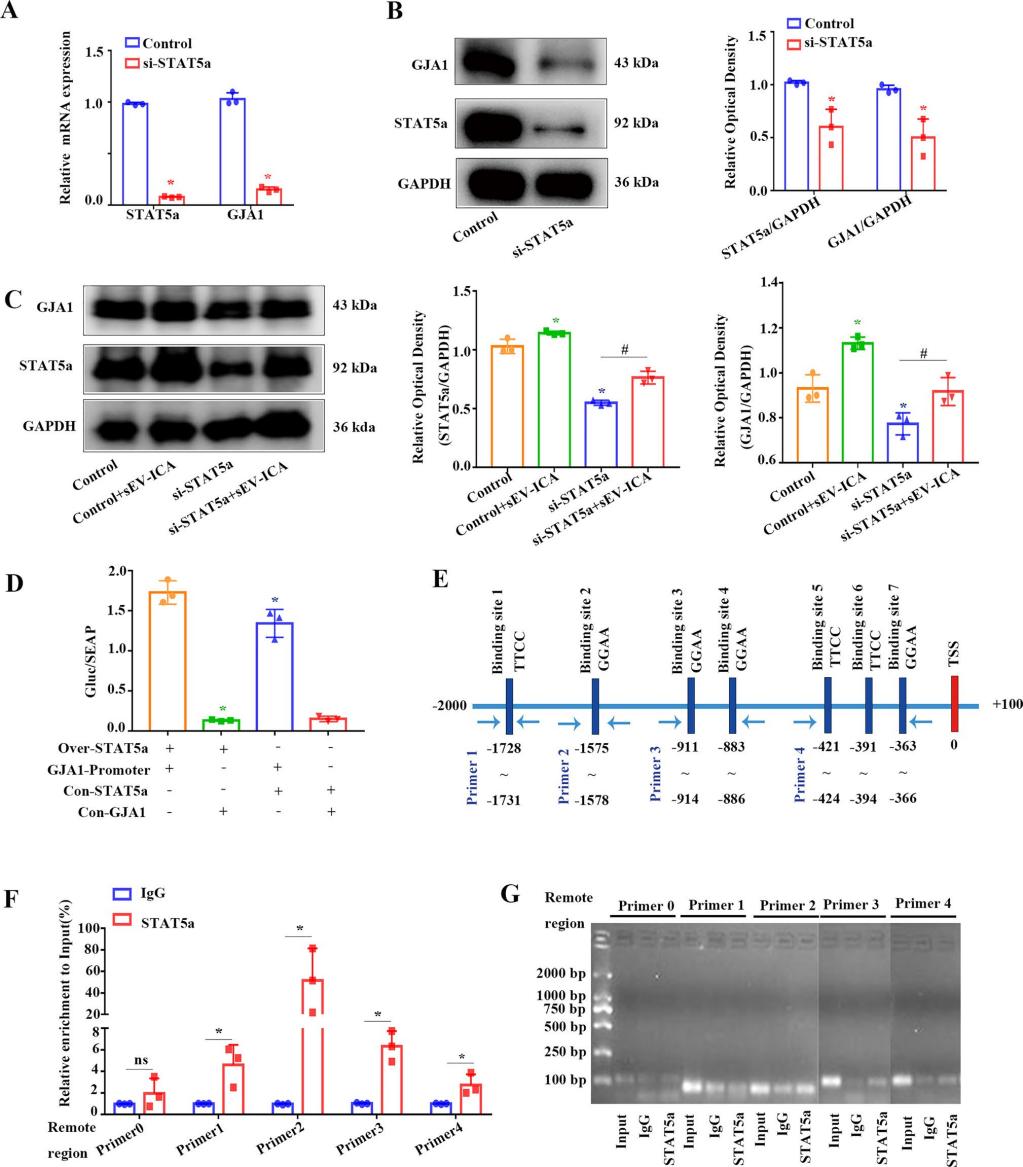
sEV-ICA promotes the binding of STAT5a to the promoter of GJA1 and strengthens expression of GJA1. (A) Real-time qPCR analysis showed the expression of GJA1 after down-regulation of STAT5a, PAX6 and Nrf2 (n = 3). Statistical significance assessed by Mann-Whitney test, ns: no significant difference, *P-value < .05. (B) The result of western-blot showed the expression of GJA1 after down-regulation of STAT5a (n = 3). (C) Western-blot show the expression of STAT5a and GJA1 after down-regulation of STAT5a with sEV-ICA (n = 3). Statistical significance assessed by Mann-Whitney test, ^*,#,†^*P*-value < .05. (D) Dual luciferase activity detected by co-transfection of Over-STAT5a and plasmid GJA1-promoter; (+) indicates that Over-STAT5a or GJA1-promoter was added, (-) indicates no addition (n = 3). Statistical significance assessed by Mann-Whitney test, *P-value < .05. (E) Bioinformatics show the predicted binding site of STAT5a on the promoter of the GJA1 gene. (F) ChIP assay confirmed the binding site of STAT5a in the promoter region of the GJA1 gene, and the histogram shows the relative level of PCR products of four binding sites on the proximal promoter of GJA1 (n = 3). Statistical significance assessed by Mann-Whitney test, ns: no significant difference, *P-value < .05. (G) The results of agarose electrophoresis show the relative levels of PCR products of four binding sites on the proximal promoter of GJA1.

Analysis using PROMO and Cistrome databases predicted that STAT5a may bind to the GJA1 promoter and activate the transcription of GJA1. The results showed that mRNA expression of GJA1 decreased after down-regulation of STAT5a expression compared to the control (Figure 7A). Western-blot experiments further proved that GJA1 was downregulated after the downregulation of STAT5a (Figure 7B). After 48 h of treatment with sEV-ICA, the expression of STAT5a and GJA1 in the control + sEV-ICA group was significantly higher than that in the control group, whereas the expression of these proteins in the si-STAT5a group was significantly decreased, and the expression of STAT5a and GJA1 in the si-STAT5a + sEV-ICA group was downregulated. This indicated that sEV-ICA may activate GJA1 transcription by upregulating STAT5a (Figure 7C). It is possible that STAT5a may bind to the GJA1 promoter to activate the transcription of GJA1.

The sequence of the GJA1 promoter (−1,000 impulse + 100) was first cloned into the luciferase reporter vector pEZX-PG04.1, and luciferase activity was detected by co-transfection. The results showed that luciferase activity in the GJA1-Promoter + Over-STAT5a group was significantly higher than in the GJA1-Promoter group (Figure 7D). These findings suggested that the transcription factor STAT5a bound to the GJA1 promoter region and activated transcription of the GJA1 promoter. The PROMO database was used to predict STAT5a-specific binding sites on the GJA1 promoter, and it was hypothesized that there were STAT5a-specific binding sites on the GJA1 promoter (Figure 7E). Specific primers were designed, and chromatin immunoprecipitation (ChIP) was performed to determine the actual binding sites. Real-time qPCR based on immunopurified DNA fragments showed that all seven predicted STAT5a binding sites on GJA1 were larger than those of the control (Figure 7F). The PCR results of agarose gel electrophoresis provided additional evidence for the ChIP assay results (Figure 7G). The above results showed that sEV-ICA can upregulate the expression of STAT5a, which binds to the binding site on the GJA1 promoter and activates GJA1 transcription.

## 4. Discussion

In fact, there is sufficient evidence to show that ICA as a bone repair drug has potential for clinical application. It can be used to prevent bone loss caused by diabetes and induce osteogenesis in vivo and in vitro through the BMP2 pathway (Qi et al., 2019; Zhang et al., 2019). However, a pharmacokinetic study showed that the plasma and tissue concentrations of ICA were markedly low and the absorption of ICA was poor after administration (Shao et al., 2018). In recent years, scholars have frequently recommended the use of milk-derived sEVs, either as stand-alone drugs or as drug carriers. Milk-derived sEVs are highly biocompatible and have limited immunogenicity even across species (Aarts et al., 2021). As a natural endogenous nano-delivery system, bovine milk-derived exosomes can not only improve the efficacy of ICA but also avoid biocompatibility problems (Agrawal et al., 2017). The sEV isolated and purified in this study by differential ultracentrifugation was characterized by the isolated particles with an average particle size of 116.8 nm and a particle concentration of 6.9 × 10^6^ particles/ml. Large-scale ultracentrifugation is therefore feasible and promises to be a viable alternative for the industrial-scale production of intact extracellular vesicles for therapeutic applications.

The drug-loading methods for sEV mainly include co-incubation, electroporation, ultrasound, extrusion, among others (Yeo et al., 2013; Agrawal et al., 2017; Familtseva et al., 2019). Among them, co-incubation is the most widely used method, but some scholars believe that it is more suitable for hydrophobic drugs or lipophilic small molecules (Pascucci et al., 2014; Luan et al., 2017). In this study, the co-incubation method was used to facilitate the loading of the hydrophobic small-molecule drug ICA onto the sEV. Transmission electron microscopy (TEM) and NTA confirmed that sEV-ICA retained their lipid bilayer structure. It was observed that the size of sEV-ICA (124.3 nm) increased moderately compared to that of sEV (116.8 nm). These results are consistent with earlier findings, such as those of Ashish et al., who used milk exosomes to load the chemotherapeutic drug paclitaxel (PAC) by co-incubation. A moderate increase in the size of Exo-PAC (108 nm) was observed compared to that of Exo (75 nm) (Haney et al., 2015). At present, the mode of interaction between sEV and drugs via which ICA is incorporated into the lipid bilayers of sEV is not clear; it could occur by surface adsorption caused by hydrophobic interaction or by the entry of drugs into the cavity of sEV (Wang et al., 2021). The loading efficiency of ICA onto sEV, as determined by HPLC, was approximately 5%. In addition to drug-loading methods, the loading efficiency of drugs may depend on the hydrophobicity of the drug molecules. The low loading efficiency of ICA is one of the shortcomings of this approach. How drugs combine with sEV and how to improve loading efficiency are problems that we hope to solve. In the next study, we will explore how to improve the loading efficiency by changing the drug loading method, performing membrane modification, etc.

**Figure 8.**
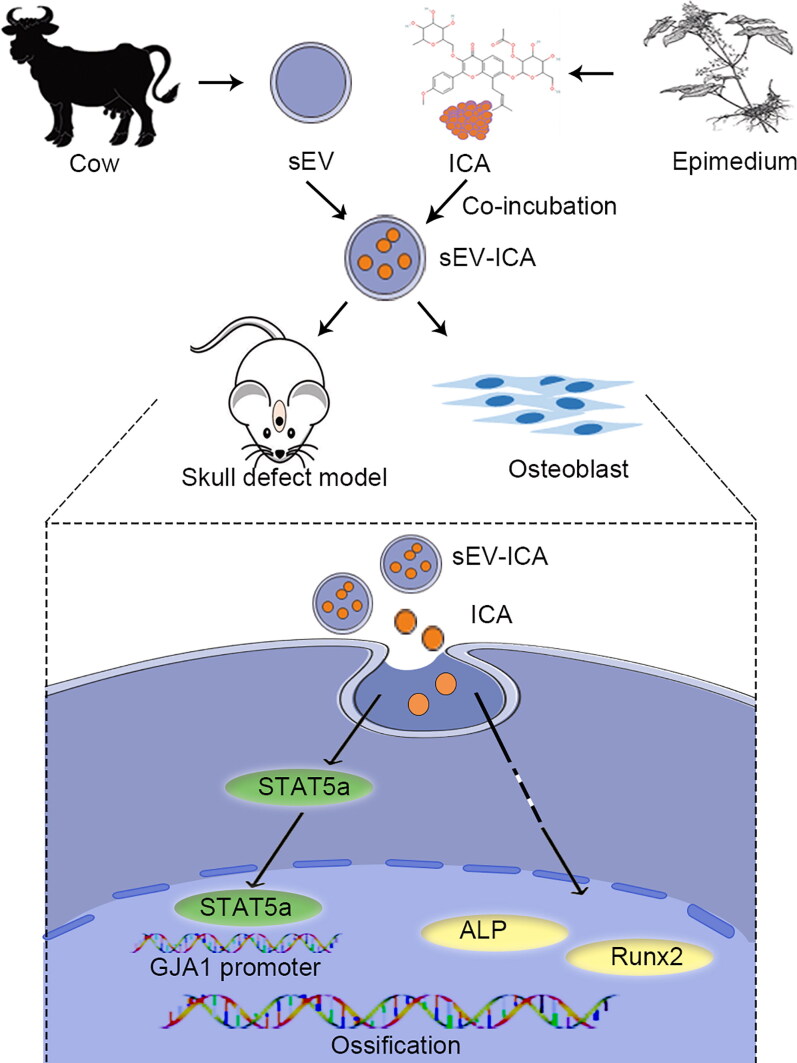
sEV-ICA can upregulate the expression of STAT5a, which binds to the binding site on the GJA1 promoter and activates GJA1 transcription.

Consistent with previous reports, we demonstrated the osteogenic effect of ICA on MC3T3-E1 cells and observed that the proliferation and differentiation of osteoblasts were further enhanced by sEV-ICA. Compared to free ICA, sEV-ICA significantly enhanced the osteogenic ability of mice skull defect model. It is believed that the enhanced ability of sEV-ICA could be attributed to the improved stability of ICA after loading ICA onto sEV, the higher uptake rate of ICA by cells, and the addition of sEV themselves because sEV alone also promote the proliferation and differentiation of osteoblasts to some extent (Masaoutis & Theocharis, 2019). Go et al. found that bovine milk sEV promoted the proliferation of human osteogenic Saos-2 cells by increasing the expression of cell cycle-related proteins. Bovine milk sEV also induced the differentiation of Saos-2 cells by increasing the expression of Runx2 and Osterix, which are key transcription factors for osteoblast differentiation. Furthermore, milk sEV promote longitudinal bone growth and increase bone mineral density in the tibia (Go et al., 2021). Bioinformatics analysis after RNA sequencing showed that the enrichment score of bone-related genes was higher in the sEV-ICA group. Through thermographic analysis of bone-related genes, we found that the expression of GJA1 was significantly higher than that in the ICA group. We also verified this result by western-blot, which suggested that GJA1 may be a key factor. GJA1 is a key regulator of bone growth and homeostasis in vivo. Through its direct effects on osteoblasts, including altering the production of osteoclast cytokines, GJA1 contributes to the acquisition of peak bone mass, cortical modeling of long bones, and maintenance of bone quality (Jiang & Cherian, 2003; Plotkin et al., 2015; Plotkin & Stains, 2015; Stains & Civitelli, 2016). Here, it was further clarified that the regulatory role of sEV-ICA on GJA1 expression involves the transcription factor STAT5a. Western-blot analysis revealed that the expression of GJA1 was downregulated after downregulation of STAT5a, whereas the expression of both increased under the action of sEV-ICA. STAT5a specifically binds to the promoter region of GJA1 to regulate the expression of GJA1 (Figure 8).

In summary, we proved that compared with ICA, sEV-ICA showed a better effect of promoting bone repair in vivo and in vitro. In addition, sEV-ICA could promote osteogenesis by promoting the combination of STAT5a and GJA1 promoter. It is suggested that sEV-ICA may be a more economical and effective method for the treatment of bone defects.

## Data Availability

The transcriptome-sequencing data is available from Sequence Read Archive (http://www.ncbi.nlm.nih.gov/bioproject/836700).
